# Safeguard against DNA sensing: the role of TREX1 in HIV-1 infection and autoimmune diseases

**DOI:** 10.3389/fmicb.2014.00193

**Published:** 2014-04-30

**Authors:** Maroof Hasan, Nan Yan

**Affiliations:** ^1^Department of Internal Medicine, University of Texas Southwestern Medical CenterDallas, TX, USA; ^2^DDepartment of Microbiology, University of Texas Southwestern Medical CenterDallas, TX, USA

**Keywords:** HIV, innate immunity, DNA sensing, Trex1, autoimmune diseases

## Abstract

Innate immune recognition is crucial for host responses against viral infections, including infection by human immunodeficiency virus 1 (HIV-1). Human cells detect such invading pathogens with a collection of pattern recognition receptors that activate the production of antiviral proteins, such as the cytokine interferon-type I, to initiate antiviral responses immediately as well as the adaptive immune response for long-term protection. To establish infection in the host, many viruses have thus evolved strategies for subversion of these mechanisms of innate immunity. For example, acute infection by HIV-1 and other retroviruses have long been thought to be non-immunogenic, signifying suppression of host defenses by these pathogens. Studies in the past few years have begun to uncover a multifaceted scheme of how HIV-1 evades innate immune detection, especially of its DNA, by exploiting host proteins. This review will discuss the host mechanisms of HIV-1 DNA sensing and viral immune evasion, with a particular focus on TREX1, three prime repair exonuclease 1, a host 3′ exonuclease (also known as DNase III).

## THE INTERFERON RESPONSE TO HIV DNA

Human immunodeficiency virus (HIV) enters T cells and macrophages by first interacting with host receptor CD4 then with co-receptor chemokine (C-C motif) receptor 5 (CCR5) or chemokine (C-X-C motif) receptor 4 (CXCR4) on the target cell plasma membrane, triggering viral envelope fusion. HIV can also bind to cell surface lectins (sugar-binding proteins) and enter cells by endocytosis. This is the predominant mode of entry into dendritic cells (DCs), which play important roles in progression and pathology of HIV infection (Luban, 2012; Manches et al., 2014), although HIV-1 does not replicate efficiently in DCs. Regardless of the mode of viral entry, the viral core containing its RNA is released into the cytosol, and HIV-encoded reverse transcriptase begins to convert viral RNA into DNA while still encapsulated in the capsid core. After completion of reverse transcription, viral integrase binds to both ends of full-length HIV-1 DNA to form pre-integration complex, which delivers functional HIV DNA to the host nucleus for chromosomal integration (Goff, 2007). Since only a few copies of HIV DNA integrate, the bulk of HIV DNA remains in the cytosol unless cleared by host enzymes (Yan et al., 2010).

**FIGURE 1:**
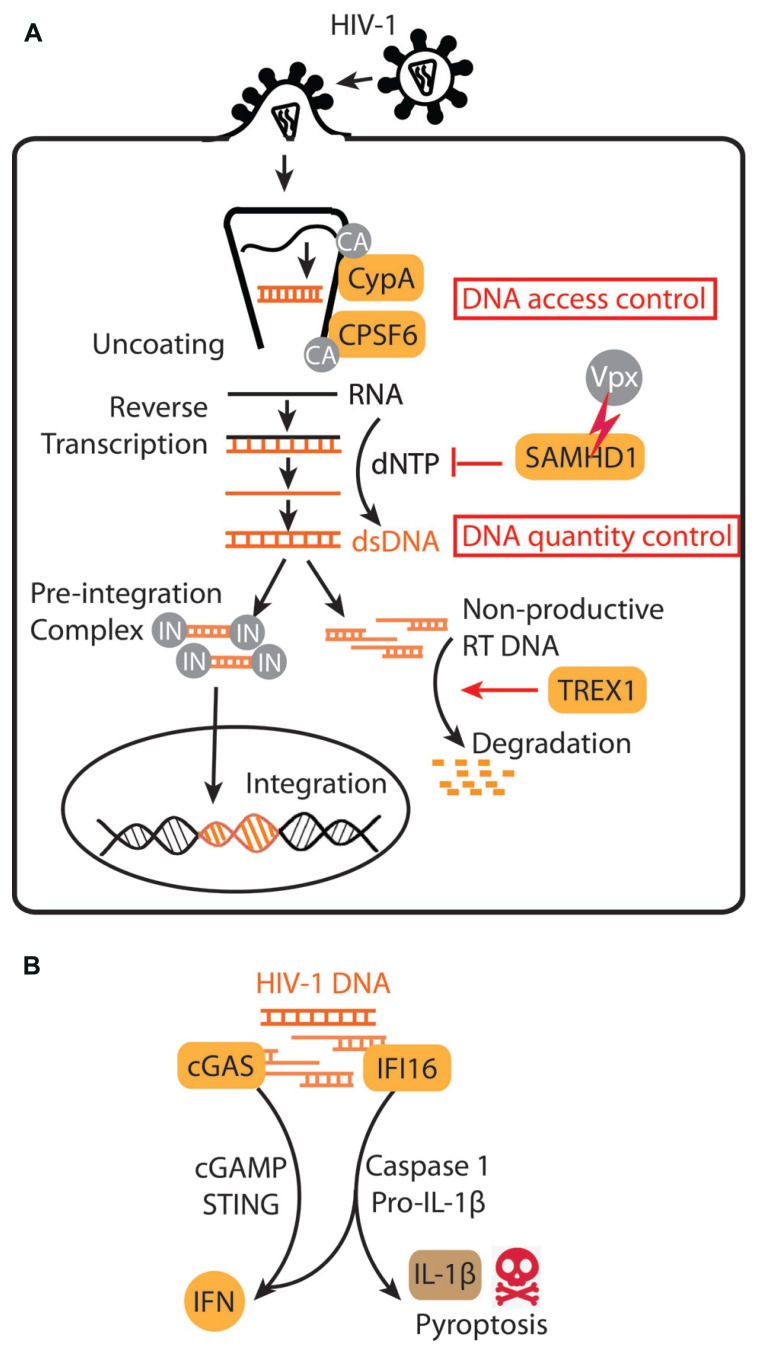
**HIV-1 subversion of innate immune DNA sensing. (A)** A schematic diagram of early stages of HIV-1 life cycle. HIV-1 capsid (CA) and associated host factors, CypA and CPSF6, regulate HIV DNA access to innate immune sensing. TREX1 and SAMHD1 regulate HIV DNA quantity. IN, integrase. **(B)** HIV-1 DNA sensing by cGAS in many target cells activates a STING-mediated IFN response, whereas HIV-1 DNA sensing by IFI16 in “bystander” quiescent CD4+ T cells trigger IFN signaling, caspase-1 activation, IL-1β secretion and pyroptosis.

Although abundant, the HIV-encoded cytosolic DNA produced by reverse transcription does not trigger a cell-autonomous interferon (IFN) or inflammatory response in activated CD4 T cells and macrophages, its primary targets (Goldfeld et al., 1991; Yan et al., 2010). HIV achieves immune evasion in these target cells by exploiting the host DNase TREX1 (Yan et al., 2010), the most abundant exonuclease in mammalian cell (Hoss et al., 1999; Mazur and Perrino, 1999), to clear its DNA. This action of TREX1 diminishes HIV DNA products in the cytosol below the threshold of immune activation. This is strikingly demonstrated in *Trex1*-/- or knockdown cells. In the absence of TREX1, HIV infection triggers a robust type I IFN response strictly dependent upon the cytosolic DNA sensing pathway, including the endoplasmic reticulum (ER) localized adaptor stimulator of interferon genes (STING), TANK-binding kinase 1 (TBK1), and the transcription factor interferon regulatory factor 3 (IRF3) (Yan et al., 2010). The HIV-stimulated IFN response in *Trex1*-/- cells can be dampened by reverse transcriptase inhibitor (e.g., nevirapine) but not by integrase inhibitor (e.g., raltegravir), consistent with HIV DNA being the main pathogen associated molecular pattern (PAMP) detected by an innate immune sensor (Yan et al., 2010). The HIV DNA is sensed by binding to cGAMP synthase (cGAS), which then synthesizes the unique second messenger dinucleotide cyclic GMP-AMP (cGAMP) that binds to STING to activate downstream IFN signaling (Gao et al., 2013; **Figure 1A**). This “competition” between TREX1 (proviral) and cGAS (antiviral) for cytosolic DNA also applies to murine leukemia virus (MLV) and simian immunodeficiency virus (SIV; Gao et al., 2013), and likely many other retroviruses.

Thus, the absence of an immunogenic response to acute retroviral infection of target immune cells can be at least partially explained by TREX1, which is ubiquitously expressed and at high levels in immune cells (Pereira-Lopes et al., 2013), and may synergize with other host factors (see below). In one population study comparing cohorts of healthy control and HIV-positive individuals, *TREX1* polymorphisms associated with susceptibility to HIV infection (Pontillo et al., 2013), although another study using different cohorts of patients failed to detect such an association (Sironi et al., 2012), suggesting that further investigation is needed before genetic linkage is established. From an evolutionary standpoint, it is intriguing that TREX1 is only found in mammals that have co-evolved with retrovirus, suggesting that retroviruses have adapted to exploit TREX1 for survival. Such an adaptation may be particularly essential for HIV, which does not appear to encode its own factors to antagonize intracellular innate immune sensing systems like many other DNA and RNA viruses do. Indeed, these findings have engendered a new paradigm for HIV-host interactions – that HIV not only exploits many host factors for the successful completion of the life cycle (Brass et al., 2008; König et al., 2008), it also exploits several key host factors that are critical for subversion of innate immune responses in target cells (Doitsh et al., 2010, 2014; Manel et al., 2010; Yan et al., 2010).

## PROINFLAMMATORY RESPONSE TO HIV DNA

HIV DNA can also trigger a proinflammatory response in non-productively infected “bystander” CD4+ T cells and promote T cell killing (Doitsh et al., 2010). HIV replication is restricted in these “bystander” CD4+ T cells due to the action of SAM domain and HD domain containing protein 1 (SAMHD1) that depletes the dNTP pool, as well as other unknown restrictive factors (Baldauf et al., 2012). As a result, HIV replication in these cells arrests early in the reverse transcription stage, although the limited amount of DNA produced can be recognized by another cytosolic DNA sensor interferon-induciable protein 16 (IFI16) (Monroe et al., 2014; **Figure 1B**). IFI16 was initially identified as a sensor that recognizes viral DNA or exogenous double-stranded DNA (introduced by transfection) and signals via STING to activate the IFN response (Unterholzner et al., 2010). A recent study found that ‘bystander” CD4+ T cells harboring abortive HIV DNA products trigger IFI16-mediated IFN signaling and inflammasome response, including activation of caspase-1, secretion of IL-1β, and death of the host cell by pyroptosis (Doitsh et al., 2014). This series of discoveries reveals another exciting example of how HIV takes advantage of DNA sensing as well as SAMHD1 restriction. In this case, instead of avoiding DNA sensing, HIV stalls DNA replication early in the reverse transcription stage to trigger inflammation and cell death in “bystander” CD4+ T cells. Since CD4+ T cell depletion is a highly diagnostic clinical feature of AIDS, these studies raise an exciting possibility of reversing CD4+ T cell depletion by blocking the inflammasome response with caspase-1 inhibitors (Doitsh et al., 2014). Both cGAS and IFI16 sense HIV DNA, yet they seem to function in distinct cell types and lead to different consequences. Also, it was unclear why TREX1 is not able to inhibit IFI16-mediated detection of HIV DNA. Further investigation is needed to show what determines which innate immune signaling pathway HIV DNA triggers or avoids, and how that influences the overall fitness of the virus.

## QUANTITY CONTROL OF HIV DNA: TREX1 AND SAMHD1

As discussed above, the amount of HIV-1 DNA in the cytosol depends upon the rates of synthesis and degradation by two host factors (**Figure 1A**): TREX1 mediates HIV DNA degradation in several immune cell types, and SAMHD1 limits HIV-1 DNA synthesis by depleting the dNTP pool in resting CD4+ T cells as well as several other cell types of myeloid linage (Hrecka et al., 2011; Laguette et al., 2011; Baldauf et al., 2012). In DCs, SAMHD1 also prevents innate immune activation (Manel et al., 2010; Sunseri et al., 2011). SAMHD1 restriction can be overcome by treating cells with virus-like particles (VLPs) containing the SAMHD1-antagnist protein Vpx, found in SIVmac and HIV-2 viruses (Goujon et al., 2006). HIV-1, which does not encode Vpx, Vpx-deficient SIVmac, and HIV-2 all fail to replicate efficiently in DCs (Hrecka et al., 2011; Laguette et al., 2011). Detailed reviews on SAMHD1/Vpx can be found elsewhere (Daugherty and Malik, 2012; Luban, 2012; Sze et al., 2013). Here, we highlight features of SAMHD1 as in comparison with TREX1.

TREX1 is expressed in most mammalian cell types (Pereira-Lopes et al., 2013), whereas SAMHD1 expression is more restricted (Laguette et al., 2011). Since a defect in either enzyme triggers HIV-1 DNA sensing, they are thought to play non-redundant roles. In cell culture, TREX1 deficiency leads to accumulation of total HIV-1 DNA, but not integrated proviral DNA (Yan et al., 2010). It therefore appears that TREX1 does not degrade full-length integration-competent HIV-1 DNA, perhaps because it is protected by integrase in the pre-integration complex. In contrast, TREX1 appears able to degrade non-productive partial-length DNA generated by error-prone reverse transcription that are not incorporated into integrase complexes. This is consistent with enzymatic properties of TREX1, a 3′ to 5′ exonuclease. Although it has some activity toward any form of DNA, it is most efficient with single-stranded DNA or double-stranded DNA with a single-stranded overhang (Mazur and Perrino, 1999). Consequently, overall HIV-1 replication in which TREX1 expression is suppressed or ablated is reduced compared replication in normal cells, because DNA sensing results in expression of antiviral IFNs (Yan et al., 2010). In contrast, SAMHD1 depletion results in increased HIV DNA synthesis, integration and overall replication (Hrecka et al., 2011; Laguette et al., 2011). It appears confusing at first glance that although both TREX1 and SAMHD1 control the quantity of cytosolic HIV DNA, the overall outcome of HIV-1 replication is quite the opposite. Also, both proteins attenuate autoimmunity, and patients carrying defective TREX1 or SAMHD1 develop similar autoimmune and sterile inflammatory phenotypes (Crow et al., 2006; Crow and Rehwinkel, 2009; Rice et al., 2009). Therefore, important questions remain. Is the antiviral role of SAMHD1 in HIV-1 pathogenesis in the complex human immune setting different from current understanding of its role as a restrictive factor? Has HIV-1 evolved to exploit SAMHD1 restriction to prevent replication in certain cell types, such as DCs, in order to avoid cell-autonomous innate immune activation, or to abort the reverse transcription stage in “bystander” CD4+ T cells to activate inflammasomes and trigger pyroptosis?

Comparison of HIV-1 with HIV-2 offers some insights into these questions. HIV-2 (containing Vpx) is far less pathogenic compared to HIV-1 (lacking Vpx) in humans, so one possible contributing factor to HIV-1 pathogenesis could be its inability to antagonize SAMHD1 or ability to avoid SAMHD1 expressing cells. One should also consider other important differences between HIV-1 and HIV-2, such as Vpu, which is only encoded by HIV-1, that antagonizes restriction factor Tetherin (Neil et al., 2008). Taken together, the opposing influences of TREX1 and SAMHD1 upon overall HIV-1 fitness suggests that it will be important to further evaluate these immunomodulators in combination as well as in isolation in immune systems.

## ACCESS CONTROL OF HIV DNA: CAPSID AND ASSOCIATED HOST FACTORS

Aside from quantity, another factor in HIV-1 DNA sensing is encapsulation of the DNA by the viral capsid, which encapsulates the incoming genomic RNA and, later on, the reverse transcribed DNA. It remains unclear whether or not the HIV-1 capsid core disassembles during or after reverse transcription, and where in the cell it occurs. In any case, capsid core uncoating and reverse transcription are closely related. Several capsid assembly mutants affect reverse transcription (Tang et al., 2001; Ambrose et al., 2012). Conversely, inhibition of reverse transcription increases stability of the HIV-1 capsid core (Yang et al., 2013). Therefore, stability of the capsid core may play a role in HIV-1 DNA access or sensing. In the case of MLV, the glycosylated Gag protein (glyco-Gag) from the incoming virus enhances viral core stability and reduces DNA sensing in TREX1 knock-down cells, so a similar relationship may apply for HIV-1 (Stavrou et al., 2013). Moreover, host cofactor proteins that bind the capsid also modulate sensing of cytosolic HIV DNA. In one study (Lahaye et al., 2013), mutations of HIV-1 and HIV-2 capsid proteins that enhance cyclophilin A (CypA) binding triggered more robust DNA sensing in monocyte-derived dendritic cells (MDDCs). In another study (Rasaiyaah et al., 2013), mutations N74D and P90A in the HIV-1 capsid protein impaired interaction with host factors cofactors cleavage and polyadenylation specificity factor sub-unit 6 (CPSF6) and CypA, and also promoted more DNA sensing in monocyte-derived macrophages (MDMs). Although different cell types were examined, both studies revealed an unexpected new dimension in sensing of HIV DNA by the host – access to the viral DNA dependent upon the viral capsid and factors encoded by the host. It remains to be determined whether the incoming capsid core simply “shields” HIV DNA from cytosolic sensing machinery by physical or steric means, or whether a more complex mechanism might be in play that regulates the dynamic connections of uncoating, reverse transcription, and nuclear import.

## SELF-DNA SENSING AND AUTOIMMUNE DISEASE

Remarkable similarity in clinical conditions between infectious and autoimmune diseases has been recognized for many years. And yet, molecular mechanisms are much better defined in infectious diseases, whereas cell-intrinsic causes of autoimmune disease remain largely mysterious. TREX1 is an excellent example of a protein that may bridge this gap in our knowledge by playing important roles in both infectious and autoimmune diseases. In addition to its role in innate immunity against HIV discussed above, TREX1 is also a critical suppressor of self-recognition that safeguards the host from erroneous autoimmune activation. Mutations in *TREX1* in humans are associated with the autoimmune and autoinflammatory disorders (Kavanagh et al., 2008; Crow and Rehwinkel, 2009) Aicardi-Goutières syndrome (AGS), familial chilblain lupus (FCL), systemic lupus erythematosus (SLE), and retinal vasculopathy with cerebral leukodystrophy (RVCL). In fact, *TREX1* represents one of the highest monogenic linkages of SLE (Moser et al., 2009). *Trex1*–/– mice develop multiple-organ inflammation, as well as autoantibodies, and succumb to inflammation burden early in age (Gall et al., 2012). Self-DNA from endogenous retroelements (Stetson et al., 2008) or DNA replication debris (Yang et al., 2007) are among the likely causes of inflammation in *Trex1*–/– mice. Inflammatory phenotypes can be rescued by eliminating essential components of the known DNA sensing pathway (e.g., *Irf3*–/–, *Sting*–/–), functionally linking the DNase function of TREX1 to disease (Stetson et al., 2008; Gall et al., 2012). Moreover, chemically modified self-DNA that resists degradation by TREX1 can cause immune activation even in the presence of TREX1. One example is oxidized DNA that contains 8-hydroxyguanosin (8-OHG), which can be formed in UV-exposed skin lesions or neutrophil extracellular traps (networks of extracellular fibers composed of mostly DNA from neutrophils). The oxidized DNA elevates cGAS/STING-mediated immune activation compared to unmodified DNA in a variety of cell types (Gehrke et al., 2013).

## TREX1: BEYOND DNase FUNCTION

TREX1 mutations that disrupt its DNase activity were mostly found in AGS, and many disease-associated mutations of TREX1 do not affect its DNase activity, especially the ones associated with SLE and RVCL (Lee-Kirsch et al., 2007; Richards et al., 2007). TREX1 is a single exon gene that encodes an exonuclease domain at its amino terminus, and an ER localization domain at its carboxyl terminus. The ER localization domain consists of a hydrophilic linker region of unknown function [although this region harbors many SLE mutations (Crow and Rehwinkel, 2009)] and a small hydrophobic segment at the extreme carboxyl-terminus that sorts TREX1 to the cytosolic leaflet of the ER membrane by serving as an ER tail-anchor (rather than a classical transmembrane span; Lee-Kirsch et al., 2007; Lindahl et al., 2009). In overexpression studies, the C-terminal domain of TREX1 mediated interaction with ubiquilin-1, leading to monoubiquitination at multiple lysine residues of TREX1 (Orebaugh et al., 2013). These modifications did not lead to TREX1 degradation, rather, they are possibly able to regulate TREX1 function or localization (Hicke, 2001). Notably, several disease-causing mutations of TREX1 exhibit altered ubiquitination patterns when co-expressed with ubiquitin (Orebaugh et al., 2013), but do not affect DNase activity. Clearly, the biological significance of monoubiquitination on native or disease-causing forms of TREX1 for viral DNA sensing and autoimmunity requires further investigation.

Abnormal accumulation of self-DNA likely promotes TREX1 AGS, since these alleles impair DNase activity, but the molecular cause for TREX1 SLE that is strongly associated with mutations in the C-terminal localization domain rather than the DNase domain remains unclear. We recently found that TREX1-deficient cells have expanded lysosomal compartments, and that TREX1 deficiency promotes lysosomal biogenesis through mTORC1 and transcription factor TFEB (Hasan et al., 2013). It remains to be seen whether this new function of TREX1 is dependent upon its DNase activity, and how it relates to various autoimmune diseases associated with *TREX1*. Interestingly, although arising from the same genetic locus, clinical conditions of TREX1 AGS and TREX1 SLE are highly distinct. TREX1 AGS mutations are autosomal recessive. Patients develop severe neurological brain diseases with excess IFNα in cerebral spinal fluid resembling intrauterine infections, affecting mostly infant and young children, many of whom die early in age (disease onset usually by 4 months; Kavanagh et al., 2008). In contrast, TREX1 SLE mutations are mostly autosomal dominant, with two exceptions where compound heterozygous missense mutations were found (Lee-Kirsch et al., 2007). Unlike TREX1 AGS, which is a pediatric disorder, TREX1 SLE disease onset is usually between 15–40 years of age as is typical for lupus-type autoimmune disorders. Similarly, TREX1 SLE patients have high titers of a wide array of autoantibodies and IFN signatures (Kavanagh et al., 2008). Despite some phenotypic overlap between SLE and AGS, distinct disease mechanisms are therefore likely.

## CONCLUSIONS AND PERSPECTIVES

Extensive effort in the past two decades provided us with incredible details on HIV-1 virology, life cycle, interactions with host factors, and antagonism of host intrinsic restriction factors with accessory proteins (Goff, 2007). In more recent years, with the rapidly expanded understanding of innate immunity, studies have begun to uncover mechanistic details of how HIV affects host innate immunity (Luban, 2012). Like many other viruses, evasion of host innate immunity is one of the most essential requirements for overall fitness of HIV-1. However, what is remarkable about HIV-1 compared to other DNA or RNA viruses is how it achieves immune evasion by exploiting host proteins. Most DNA or RNA viruses encode viral proteins that target specific intracellular innate immune sensing pathways involved in recognizing DNA or RNA, or immune activation. HIV-1 does it by exploiting host negative regulators of innate immunity to subvert sensing of its own DNA. The focus of this review, DNase III/TREX1, normally safeguards the host from autoimmune activation by self-DNA, but is exploited by HIV-1 to evade sensing of its own DNA. In a seemingly counterintuitive move, HIV-1 does not encode Vpx that antagonizes a potent restriction factor SAMHD1. Interestingly, *vpx* deletion during the evolution of SIVcpz (that eventually gave rise to HIV-1) resulted in the creation of a unique *vif* that can antagonize hominid restriction factor APOBEC3 (Etienne et al., 2013). Whether this curious omission of Vpx contributes to overall HIV-1 fitness, and how much of that advantage is contributed from SAMHD1, remain to be seen. This could be an unprecedented way for a virus to subvert immune activation – by keeping its replication low in professional IFN producing cells such as DCs. Along the same line, abortive HIV DNA products in bystander quiescent CD4+ T cells (due to the SAMHD1 block) activate IFI16-mediated inflammasome response and pyroptosis – an effective way to paralyze the host immune system.

These discoveries of unique HIV immune evasion strategies may mark the beginning of an exciting new era on studies of HIV innate immunity, with important new avenues to be explored. Further studies in this emerging field will certainly open our eyes on how HIV navigates the human innate immune system. This knowledge could also be harnessed for novel strategies of HIV vaccine design that specifically target HIV immune evasion. We look forward to seeing these studies unfold.

## Conflict of Interest Statement

The authors declare that the research was conducted in the absence of any commercial or financial relationships that could be construed as a potential conflict of interest.
